# Endoplasmic Reticulum Protein ERp46 in Renal Cell Carcinoma

**DOI:** 10.1371/journal.pone.0090389

**Published:** 2014-03-03

**Authors:** Wilhelmina C. M. Duivenvoorden, Athanasios Paschos, Sarah N. Hopmans, Richard C. Austin, Jehonathan H. Pinthus

**Affiliations:** Department of Surgery, McMaster University and St. Joseph's Healthcare Hamilton, Hamilton, Ontario, Canada; University of Kentucky College of Medicine, United States of America

## Abstract

An established inverse clinical correlation between serum adiponectin levels and renal cell carcinoma (RCC) aggressiveness exists. We have recently demonstrated that adiponectin suppresses clear cell RCC (ccRCC) progression through interaction with its receptor, adiponectin receptor 1 (AdipoR1). ERp46 has been shown to inhibit adiponectin signaling via interaction with AdipoR1 in HeLa cells. However, the expression of ERp46 in RCC has not been described thus far. The objectives of this study were to investigate ERp46 in RCC, its expression, its effects on RCC growth in a mouse model and whether it interacts with AdipoR1. We demonstrated a higher ERp46/AdipoR1 expression ratio in metastatic compared to non-metastatic ccRCC, as determined by immunohistochemistry of tissue microarrays and subsequent image analysis. When ERp46 was stably knocked down using shRNA or overexpressed in murine RCC RAG cells, RCC growth after subcutaneous injection in BALB/c nude mice was inhibited and accelerated, respectively. *In vitro* analysis to determine the molecular interaction between AdipoR1 and ERp46 included co-immunoprecipitation using human ccRCC 786-O cells and a bacterial adenylate cyclase-based two hybrid system and demonstrated no sustained AdipoR1-ERp46 interaction. This is the first report to suggest a role for ERp46 as a potential therapeutic target in RCC given its expression profile in human RCC samples and its effect on *in vivo* RCC growth. Since a stable interaction with AdipoR1 could not be established, we suggest that the tumorigenic properties of ERp46 in RCC cells are not related to an inhibitory modulation of AdipoR1.

## Introduction

Altered levels of fat tissue-derived hormones in obesity, an established risk factor for renal cell carcinoma (RCC) [1], can potentiate the growth of different cancers. Obesity is associated with reduced circulating levels of adiponectin [2]–[4]. Clinically, we [5] as well as others [6] found an inverse correlation between serum/plasma adiponectin levels and RCC aggressiveness. Our previous studies have also shown that adiponectin inhibits the angiogenic, invasive and migratory capacities in clear cell RCC (ccRCC) cells via adiponectin receptor 1 (AdipoR1) [7] and that these tumor-suppressive effects are diminished in RCC not only due to decreased plasma adiponectin levels [5], but also as a result of reduced AdipoR1 expression [7]. We therefore hypothesize that maximizing AdipoR1 signaling may be a novel therapeutic approach for RCC. Maximizing tumor suppressive signaling may be achieved by targeting its negative regulators [8], [9]. It has recently been demonstrated in Chinese hamster ovary cells, that endoplasmic reticulum (ER) protein ERp46, a member of the protein disulphide isomerase (PDI) family of oxidoreductases [10], binds to recombinant human Flag-tagged AdipoR1 and that knockdown of ERp46 leads to an increase in activation of AMPK in HeLa cells [11]. Whether this interaction occurs under native conditions and is responsible for enhancing cancer cell proliferation remains elusive. The expression and function of ERp46 in RCC has not been studied. Using different methodologies both *in vitro* and *in vivo*, we explored the suitability of ERp46 as a potential therapeutic target in RCC. In this study, we demonstrate a significantly higher ERp46/AdipoR1 expression ratio in metastatic ccRCC compared to non-metastatic ccRCC and show that increased expression of ERp46 promotes RCC growth *in vivo*. However, the tumorigenic properties of ERp46 in RCC cells are not likely related to an inhibitory modulation of adiponectin's tumor-suppressive signaling, as an interaction with AdipoR1 could not be established.

## Materials and Methods

### Ethics statement

The animal study was carried out in strict accordance with the guidelines of the Canadian Council of Animal Care and was reviewed and approved by the McMaster University Animal Research Ethics Board (protocol #12-12-42). All necessary steps were taken to minimize suffering and distress to the mice.

### Strains, plasmids, cell lines and cell culture

*Escherichia coli* strains DH5α [12] and BTH101 [13], [14] were used as hosts for cloning and protein overproduction, respectively. Plasmids used are listed in Table S1. Human ccRCC 786-O and murine RCC RAG cells were obtained from ATCC. 786-O cells were propagated in RPMI-1640 medium supplemented with 10% (v/v) fetal bovine serum (FBS, Life Technologies Inc.), the RAG cells in MEM supplemented with 10% FBS. Cells were cultured in a humidified atmosphere at 5% CO_2_ and 37°C. The cells were routinely verified to be mycoplasma-free and the identity of the human 786-O cell line was verified by STR analysis (ATCC).

### Transfection and short-hairpin RNA

The shRNA vector for ERp46 (ERp46 shRNA), the non-effective negative scrambled control and pCMV6-Kan/Neo plasmid containing the full-length cDNA encoding for murine ERp46 were purchased from Origene. Transfection of murine RAG cells was performed using Lipofectamine (Life Technologies Inc.). Cells stably transfected with shERp46 or scrambled control were selected with 0.5 µg/ml puromycin, cells with full-length ERp46 or empty vector with 0.9 mg/ml G418.

### Plasmid construction

Plasmid pKT25-AdipoR1_N_, pUT18C-ERp46_N_, pUT18C-L-ERp46_N_, and pKTN25-AdipoR1_N_ were constructed to express the N-terminal AdipoR1 and ERp46 fragments (see Table S2) fused to the C-termini of T18 and T25 under the control of the *lacZ* promoter in kanamycin or ampicillin-resistance-determining vectors, pKT25 and pKTN25, and pUT18C, respectively [13], [14]. To this end, a *Pst*I-*Kpn*I fragment in pUT18C or pKT25 was replaced by PCR-amplified AdipoR1 or ERp46 N-terminal DNA sequences flanked by the same restriction sites. In the case of ERp46, a variant with the inclusion of a *N*-GGSGLVGGSGGGSGGGSGGGSGGGSGGGSGGGST-*C* linker was additionally constructed. Initially a *PstI-XbaI* DNA-linker containing sequence was cloned in pUT18C, and subsequently a *Xba*I-*Kpn*I fragment containing the ERp46-N-terminus in pUT18C. Amplification of AdipoR1 was performed using a standard PCR protocol, and the parental plasmid pDONR223-AdipoR1 served as template for the N-terminal AdipoR1 cDNA. N-terminal ERp46 fragments were chemically synthesized (Mobix, McMaster University).

### Growth of bacterial cultures and reporter enzyme assay

The quantification of the functional complementation mediated by interaction between two proteins was obtained by measuring β-galactosidase activity. The appropriate plasmids were transferred in BTH101 *Escherichia coli* cells [13], [14]. Single colonies were picked and inoculated in 2 ml LB medium (1% casein hydrolysate, 0.5% yeast extract, 1% NaCl). Plasmid-carrying BTH101 cells were cultured in 2 ml LB medium supplemented with the appropriate antibiotics (ampicillin, 150 µg/ml or kanamycin, 50 µg/ml) and 1 mM isopropyl-β-d-thiogalactopyranoside to induce expression of the T18 and T25 fusion proteins. After 18 h growth, the protein interaction was colorimetrically assessed by cAMP-dependent galactosidase activity by incubation of 20 µl of culture with 80 µl Z buffer (0.06 M Na_2_HPO_4_·7H_2_O, 0.04 M NaH_2_PO_4_·H_2_O, 0.01 M RCCl, 0.001 M MgSO_4_) containing 8 mg/ml 2-nitrophenyl-β-d-galactopyranoside, 0.01% SDS, and 50 mM β-mercaptoethanol. The reaction mixtures were incubated for 0.5 h to 2 h at room temperature, and the reactions were stopped with 100 µl 1 M Na_2_CO_3_. The endproducts were measured at 420 nm and 550 nm with a BioTek Powerwave HT plate reader. Specific activity was calculated as follows: Miller units  =  [OD_420_ − (1.75×OD_550_)]/[*t*×OD_600_× (volume in ml)]×1,000, where OD_600_ is the optical density at 600 nm after 18 h of incubation and *t* is the time needed for color formation.

### Immunocytochemistry, immunohistochemistry and image analysis

Human ccRCC 786-O cells were grown to subconfluency on glass coverslips and fixed using 2% paraformaldehyde in PBS for 30 min. Immunocytochemistry was performed using mouse polyclonal ERp46 antibody (12 µg/ml, Sigma) followed by anti-mouse Alexa 488-labelled antibody (1∶500, Life Technologies Inc.), or AdipoR1 antibody (6 µg/mL, Phoenix Pharmaceuticals) followed by anti-rabbit Alexa 594-labelled antibody (1∶500, Life Technologies Inc.). Nuclei were counterstained using DAPI (4′,6-diamidino-2-phenylindole, 3 µM in PBS).

A tissue microarray (TMA) with duplicate cores from a total of 23 ccRCC, 8 normal kidney specimens, and 9 specimens from RCC metastatic sites were obtained from US Biomax (KD951). Immunohistochemistry for AdipoR1 was performed as described in [7]. Immunostaining for ERp46 was performed using heat-induced antigen retrieval in pre-heated citric acid buffer (10 mM, pH = 6.0) for 30 min, and mouse polyclonal anti-ERp46 antibody (3 µg/ml, Sigma) overnight at 4°C, followed by a 60 min incubation with anti-mouse HRP-labeled polymer (Dako). Immunohistochemical staining controls included human lymph node (positive control) and by omission of primary antibody (negative control). The immunostained TMA was digitized on AperioScan XT (Aperio Technologies Inc.). Using the ImageScope software (version 11.1.2.760, Aperio Technologies Inc.) and the positive pixel count (v9) algorithm provided, the H-score for the carcinoma or kidney tissue within each core of the TMA was determined as a measure of staining intensity according to [15]. For each individual, the adjusted H-score was calculated by subtracting the H-score of the negative control and averaging the duplicate H-scores. The AdipoR1 and ERp46 protein expression in the cancer was normalized to their expression in normal tissue and the ratio of ERp46/AdipoR1 protein expression in specimens of ccRCC patients was determined.

### Western blot analysis and co-immunoprecipitation

Cell lysates (30 µg) were resolved on a 12% SDS-PAGE gel and proteins were transferred onto a PVDF membrane. The following primary antibodies were employed: goat anti-ERp46 (1∶1,000; Santa-Cruz), rabbit anti-AdipoR1 (1∶1,000; AbBiotech), rabbit anti-HDAC2 (1∶1500; Santa-Cruz), rabbit anti-Hsp90 (1∶1,000; Cell Signalling), rabbit anti-calreticulin (1∶1,000; Cell Signaling). Secondary antibodies conjugated with HRP (1∶3,000; Jackson ImmunoResearch Laboratories) were used in conjunction with chemiluminescence detection. An antibody specific for β-actin (1∶1,000, Sigma-Aldrich) served as internal control.

For co-immunoprecipitation, protein lysates were prepared from subconfluent 786-O cells by cryolysis using 20 mM Potassium-HEPES buffer (pH = 7.4). An antibody against AdipoR1 (AbBiotech) covalently bound to magnetic Dynabeads (Life Technologies Inc.) was used for precipitation, while an antibody against ERp46 (Santa Cruz) was used for detection. Buffers used for co-immunoprecipitation consisted of the 1x IP buffer provided by the Dynabead kit (Life Technologies Inc.) with or without modifications (see Table 1).

**Table 1:** Different buffer conditions used in the co-immunoprecipitation experiments and the interaction status of ERp46 and AdipoR1 found.

Buffer Condition	Interaction status
Buffer A (1X IP buffer (containing 110 mM KOAc, 0.5% Triton X100) plus 100 mM NaCl).	No
Buffer B (1X IP buffer plus 100 mM NaCl, 2 mM MgCl_2_, 1 mM DTT)	No
Buffer C (1X IP buffer plus 50 mM NaCl, 1 mM MgCl_2_, 0.5 mM DTT)	+/−
PBS plus 1 mM MgCl_2_, 1 mM CaCl_2_	+

### Analysis of protein–protein interactions by crosslinking

Chemical crosslinking was performed by enriching cell lysates from 10^5^ 786-O cells with crosslinking reagents (disuccinimidyl suberate (DSS), bis[sulfosuccinimidyl] suberate (BS3), bismaleimidoethane (BMOE), and formaldehyde) to a final concentration of 1 mM. The mixtures were incubated at room temperature for 1 h. If applicable, DTT was added at a concentration of 1 mM. Thirteen µl of the reaction was analyzed by SDS/PAGE and subsequent Western blotting.

### Subcellular fractionation and surface protein isolation

Subcellular fractionation and surface protein isolation were carried out using subcellular protein fractionation and cell surface protein isolation kits (Pierce, Thermo Fisher Scientific Inc.), respectively, following the manufacturers' instructions. We chose a commercially available cell fractionation kit showing a favorable and time-efficient separation of cytoplasmic, membrane and nuclear fractions [16]. Briefly, a stepwise separation of cellular compartments from 3×10^6^ human ccRCC 786-O cells was performed after application of the supplied cytoplasmic, membrane, nuclear soluble, chromatin-bound and cytoskeletal protein extraction buffers. For the cell surface protein isolation, biotinylation of cell surface proteins with EZ-Link Sulfo-NHS-SS-biotin was performed using 3×10^6^ human ccRCC 786-O cells. After cell lysis, biotinylated proteins were eluted by NeutrAvidin affinity chromatography.

### Animal studies

Per treatment group, male inbred BALB/c nude mice (Charles River, 5 weeks of age, 15-20 g) were randomly divided into groups of seven to ten mice. The mice (5/mouse microisolator cage) were kept in an ultra clean room on a 12/12 h-light/dark cycle in a temperature- and humidity-controlled environment with food (#2918 irradiated Teklad rodent diet, Harlan Laboratories,) and water available *ad libitum*. Each cage contained alphadry bedding and a rubber tube to play/sleep for enrichment. Parental murine RAG, shERp46, ERp46+, empty vector (EV) and scrambled control (shControl) RAG cells were resuspended at a concentration of 1×10^7^ cells/ml (1∶1 (v/v) serum-free medium:Matrigel (BD Biosciences)). Cell viability was >95% by trypan blue exclusion. Cells (2×10^6^ cells in 200 µL) were injected subcutaneously into the right flank of the mouse using a 1 ml syringe and a 25G needle. The size of the tumors was measured every three days using plastic Vernier calipers. The mice in all groups were measured together, alternating the cage order and by randomly selecting the mice for each cage. The tumor volume was calculated using the formula π/6 (length*width*height). After 5 weeks when the largest tumor reached 600 mm^3^, all animals were sacrificed by exposure to CO_2_, the tumors dissected and weighed, and serum collected. No adverse effects of the treatment were observed.

In a separate experiment, parental RAG cells (2×10^6^) were subcutaneously injected and the mice (n = 10/group) were randomly divided into two groups. One group was treated with shRNA specific for ERp46 using the delivery agent *in-vivo*jetPEI (PolyPlus Transfection SA) via intraperitoneal injection (150 µl) every second day with 21 µg shRNA/mouse and N/P (*in-vivo*jetPEI/DNA) ratio of 7 using a 25 G needle. Control mice were injected with scrambled control shRNA using the same protocol. The mice in the two groups were treated together, alternating the cage order and by randomly selecting the mice for each cage. The volume of the tumor in each mouse was determined every three days until the largest tumor reached 600 mm^3^. No adverse effects of the treatment were observed. After 5 weeks, the animals were sacrificed by exposure to CO_2_, the tumors dissected and weighed.

The levels of VEGF were determined in mouse serum using a mouse VEGF ELISA kit (R&D Systems, Minneapolis MN, USA) according to the manufacturer's instructions on a BioTek Powerwave HT multiwell plate reader.

Immunostaining of four µm-thick tumor xenograft sections for CD31 and subsequent image analysis were performed according to [7]. As a measure of microvessel density in the tumor tissue, two fields of view at 7x magnification with the highest vessel density were selected to determine the total cumulative linear endothelial length using the ImageScope software. The endothelial length (in µm) was divided by the area of the field of view in mm^2^.

### Statistical analysis

Values are given as the mean plus or minus 95% confidence intervals. The normally distributed data were analyzed using a two-tailed Student's t-test. A *p*-value<0.05 was regarded as significant. The longitudinal tumor volumes were analyzed using one-way ANOVA. Statistical analyses were performed using MiniTab version 14.

## Results

### Increased ERp46/AdipoR1 ratio in clinical specimens of human metastatic ccRCC

**Figure 1:**
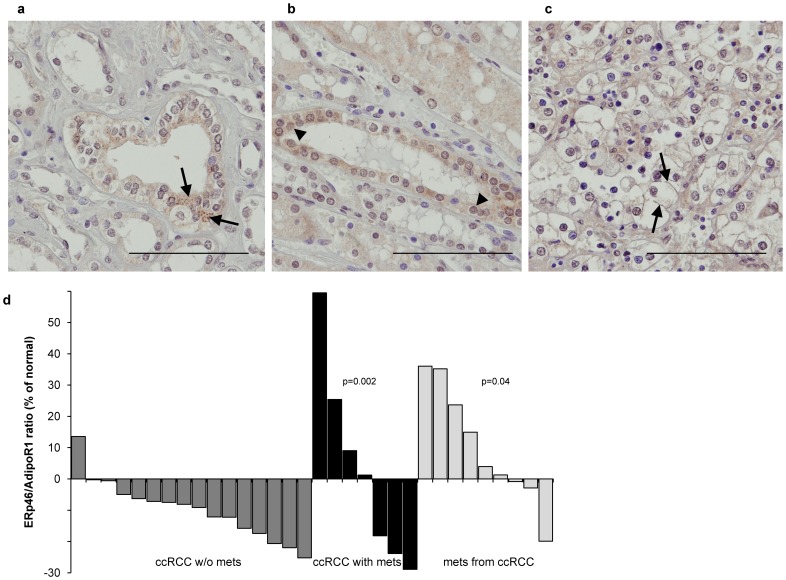
ERp46 expression is increased in metastatic human ccRCC tissue. ERp46-immunohistochemistry of human normal kidney tissue demonstrates (**a**) granular cytoplasmic staining typical for ER (examples indicated by the arrows), but also (**b**) nuclear staining (arrow heads). (**c**) ERp46-staining of human ccRCC showing plasma membrane staining (arrows). Original magnification 630x; Bar = 100 µm. (**d**) The ratio of ERp46/AdipoR1 protein expression in specimens of ccRCC patients was significantly increased in primary ccRCC from patients with metastasis (p = 0.002) and in metastatic tissue (p = 0.04). ERp46 and AdipoR1 protein expression was quantified by image analysis (H-score). The AdipoR1 and ERp46 protein expression in the cancer was normalized to their expression in normal tissue. The ratios obtained from the patients with primary ccRCC without distant metastasis are represented by the dark grey bars, the black ones represent the primary ccRCC samples from patients with metastasis, the light grey ones are from ccRCC metastatic samples.

Using ERp46-immunohistochemistry on tissue microarrays containing ccRCC samples of different stages and corresponding metastatic and normal renal specimens, we show the presence of ERp46 protein in specimens from ccRCC (Figure 1c) and in normal renal tissue (Figure 1ab). In normal kidney, ERp46 staining was prominent in the cytoplasm with a granular pattern indicative of ER staining (Figure 1a), but in some cases staining was also observed in the nucleus of the tubular epithelial cells (Figure 1b). In ccRCC, the staining appeared membranal (Figure 1c). The ERp46 protein expression was quantified by image analysis (H-score). Among the tumor specimens, RCC metastatic tumors showed the strongest ERp46 staining, followed by the primary ccRCC samples obtained from metastatic patients, and the primary ccRCC samples from patients without distant metastasis exhibiting the least staining. Since ERp46 has previously been suggested to act as a negative modulator of the adiponectin signal transduction pathway through binding to AdipoR1, but not AdipoR2 [11] and we found that AdipoR1 was the primary receptor through which adiponectin exerts its effects [7], we also determined the ratio of ERp46/AdipoR1 protein expression in specimens of ccRCC patients (Figure 1d) using the H-scores from the same TMA stained for AdipoR1 [7]. The ratio of ERp46/AdipoR1 protein was significantly increased in metastatic tissue compared to primary ccRCC from patients without metastasis (p = 0.002), but also compared to primary ccRCC from patients with and without metastasis (p = 0.04).

### ERp46 supports *in vivo* tumor growth

**Figure 2:**
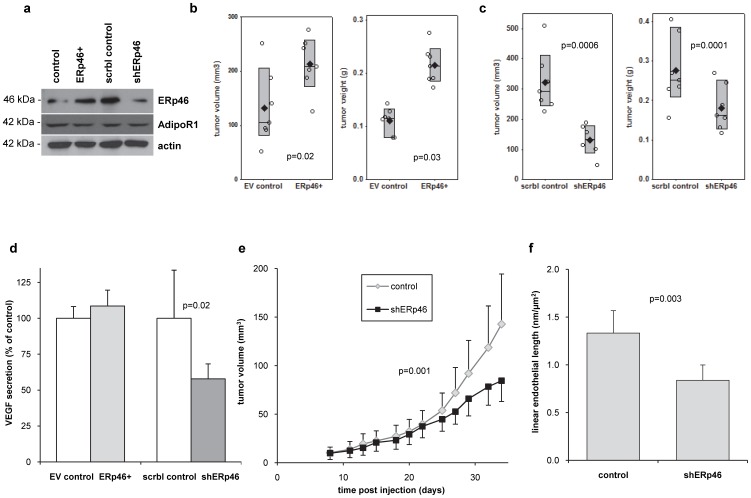
ERp46 supports *in vivo* tumor growth. (**a**) ERp46-manipulated subclones of mouse RCC RAG cells demonstrated a 4-fold increase in ERp46 protein expression in ERp46-overexpressing (ERp46+) cells, and a 80% knockdown of ERp46 protein expression in cells stably expressing shRNA specific for ERp46 (shERp46) compared to corresponding control transfected cells. There was no difference in AdipoR1 expression. ERp46 and AdipoR1 were detected by Western blot analysis. The protein expression of β-actin served as loading control. (**b**) *In vivo* growth of different ERp46-manipulated stable subclones of mouse RCC RAG cells. Tumor volume and weight from mice (n = 7/group) injected with ERp46+-RAG cells is significantly higher than from mice injected with empty vector (EV) control-transfected cells (p = 0.02 and 0.03, respectively). Individual values (○) and mean (♦) are shown, the box indicates the 95% confidence interval. (**c**) Tumor volume and weight from mice (n = 7/group) subcutaneously injected with shERp46-RAG cells is significantly lower than from mice injected with cells stably transfected with scrambled control shRNA (p = 0.0006 and 0.0001, respectively). Individual values (○) and mean (♦) are shown, the box indicates the 95% confidence interval. (**d**) Serum VEGF of the mice subcutaneously injected with ERp46-manipulated RAG cells 35 days after injection (n = 7/group). Serum VEGF is significantly lower in shERp46 RAG cell-injected mice compared to corresponding control mice (p = 0.02). Data represent mean ±95% confidence intervals. (**e**) Longitudinal tumor growth of mouse RCC RAG cells *in vivo*. Mice were subcutaneously injected with 2×10^6^ RAG cells and treated systemically (intraperitoneally) every second day with shRNA specific for ERp46 (shERp46) or scrambled control shRNA (control) (n = 10/group) using the *in-vivo*jetPEI delivery agent (p = 0.001; ANOVA). Data represent mean ±95% confidence intervals. (**f**) At sacrifice (35 days), the linear endothelial length as determined in CD31-stained subcutaneous tumors is significantly lower (p = 0.003) in shERp46-treated RCC RAG cell-injected mice (n = 10) compared to mice treated with shControl (n = 10). Data represent mean ±95% confidence intervals.

To investigate the significance of ERp46 expression on RCC, stably transfected murine RCC RAG subclones were generated. These cells expressed either 80% knockdown of ERp46 protein expression (shERp46) or a 4-fold increase in ERp46 protein expression (ERp46+) compared to the respective control cells (scrambled shRNA or empty vector transfected RAG cells) (Figure 2a). We determined the *in vivo* growth of the different ERp46-manipulated subclones of RCC RAG cells after subcutaneous injection into nude mice (n = 7/group) which achieved a tumor take rate of 100%. Western blot analysis of the tumor homogenates showed that ERp46 remained overexpressed in the ERp46+-injected mice (1.6-fold; data not shown) and reduced in the shERp46-mice (21%) at sacrifice, five weeks after tumor cell injection. The tumor volume and weight from mice injected with ERp46+ RAG cells was significantly higher than from mice injected with empty vector (EV) control-transfected cells (Figure 2b; p = 0.02 and 0.03, resp.). Tumor volume and weight from mice injected with shERp46 RAG cells was significantly lower than from mice injected with cells transfected with scrambled control shRNA (Figure 2c; p = 0.006 and 0.001, resp.). Moreover, the serum VEGF values at the time of sacrifice were also significantly lower in the shERp46-injected mice compared to the shControl-injected mice (Figure 2d; p = 0.02).

In a separate experiment, twenty mice (n = 10/group) were subcutaneously injected with parental RAG cells. Mice were treated intraperitoneally every two days either with shRNA specific to ERp46 or scrambled control shRNA using *in-vivo*-jet PEI and sacrificed five weeks later. Tumor volume from mice injected with shRNA specific for ERp46 was significantly lower than from mice injected with scrambled control shRNA (Figure 2e; p = 0.001, ANOVA). Using immunohistochemical staining of the subcutaneous tumors for CD31, an endothelial marker, we also demonstrate a decreased amount of microvessels in the shERp46-treated mice, with a linear microvessel length decrease of 37% (Figure 2f, p = 0.03).

### Subcellular localization, co-immunoprecipitation and crosslinking of ERp46 and AdipoR1

**Figure 3:**
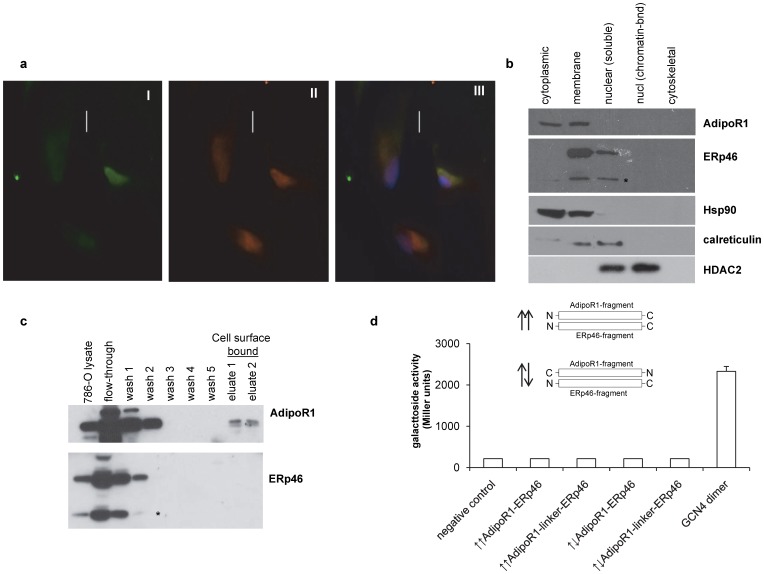
Co-localization of ERp46 and AdipoR1 in human ccRCC 786-O cells, but no interaction. (**a**) Immunocytochemical staining for ERp46 (I, green), or AdipoR1 (II, red). The merged image (III) demonstrates yellow signal which indicates co-localization. Cells were counterstained with DAPI (blue). (**b**) Subcellular protein fractionation. Equal portions of each fractionated cellular extract were analyzed by Western blot using specific antibodies against AdipoR1 and ERp46. Antibodies directed against Hsp90 (cytoplasmic), calreticulin (plasma membrane) and HDAC2 (nuclear) served as fractionation controls. AdipoR1 is detected in the cytoplasmic and plasma membrane fractions, ERp46 in the nuclear soluble and membrane fraction. The asterisk indicates an ERp46 degradation product or possibly the shorter ERp46 isoform 3. (**c**). Western blot analyses for AdipoR1 and ERp46-specific extraction and isolation from 3×10^6^ 786-O cells. Presence of AdipoR1 in the elution fraction confirms that AdipoR1 is cell surface exposed. Absence of ERp46 in the elution fraction indicates most likely that it is either not cell surface exposed or not tightly bound to a cell surface protein. (**d**) Bacterial adenylate cyclase-based two-hybrid assay (BACTH) used to determine the interaction between ERp46 and AdipoR1. The N-termini of AdipoR1 and ERp46 were expressed as fusion to the T18 and T25 domains of the adenylate cyclase. Interaction was quantified via cAMP/CAP-induced β-galactosidase activity. The pUT18C strain producing AdipoR1_N_ fused to T25 domain of adenylate cyclase served as negative control. Other controls (plasmid, ERp46_N_ with linker, ERp46_N_ fused to T18) were also negative. No interaction between the N-termini of ERp46 and AdipoR1 was observed. T18 and T25 fused to interacting leucine zippers from GCN4 served as positive control. Data are from three independent repetitions and error bars indicate standard deviation. Depending on the fusion direction of AdipoR1_N_ and ERp46_N_ to T18 and T25, a “parallel” and “anti-parallel” orientation of the N-termini is captured, shown schematically.

ERp46 has previously been suggested to be involved in adiponectin signal transduction events and to act as a negative modulator of this hormonal axis [11]. Since our data suggest that increased expression of ERp46 promotes RCC growth *in vivo* and we have previously shown that adiponectin suppresses RCC progression through the interaction with its receptor AdipoR1 [7], ERp46 seems an appealing potential therapeutic target in RCC. One way to maximize the tumor-suppressive effects of adiponectin in RCC is to target its putative negative regulator ERp46. Consequently, a closer molecular examination of the ERp46 interaction with AdipoR1 was undertaken. To interact physiologically, ERp46 and AdipoR1 need to be present in the same cellular compartment. ERp46 has been described to be primarily localized to ER [11], [17], but also in cytosolic and membrane fractions [11], [18]. AdipoR1 is a polytopic transmembrane protein found in the outer cellular membrane with its N-terminus localized internally [19], [20]. We performed immunocytochemistry for ERp46 and AdipoR1 in human ccRCC 786-O cells and observed a merged yellow signal indicative of co-localization (Figure 3a). We also carried out subcellular protein fractionation (Figure 3b) and cell surface protein extraction (Figure 3c) which show that AdipoR1 was present in the membrane and cytoplasm, whereas ERp46 was found in the membrane and partially in the nuclei. A second smaller immunodetectable ERp46 protein was also observed, likely representing a degradation product [21]. Interestingly, AdipoR1, but not ERp46, was detected at the cell surface. These observations concur with our immunohistochemical data from the clinical samples, both for ERp46 (Figure 1) and for AdipoR1 [7].

ERp46 has been described to co-immunoprecipitate with AdipoR1, after coexpression of FLAG-tagged human AdipoR1 in Chinese hamster ovary cells [11]. We performed co-immunoprecipitation in human ccRCC 786-O cell lysates using an antibody against AdipoR1 to immunoprecipitate and an ERp46-directed antibody to detect the AdipoR1-ERp46 complex. Several independent experiments using different buffer conditions led to a non-conclusive AdipoR1-ERp46 interaction status in ccRCC 786-O cells (Table 1). Increasing the ionic strength and varying the cationic ions in the immunoprecipitation buffer yielded occasionally non-reproducible positive interactions, indicating the AdipoR1-ERp46 interaction to be, if *de-facto*, potentially transient.

To further identify a potential AdipoR1-ERp46 interaction in 786-O cells, we also used several different crosslinkers as a means to visualize stable or transient protein-protein interactions [22]. Our results show changes in the migration patterns on the Western blots for AdipoR1 and ERp46 in comparison to absence of crosslinkers, however, the bands detected resembled a smear indicating the presence of at least several interacting partners in the case of both AdipoR1 and ERp46 (data not shown). Due to the lack of available purified AdipoR1 or ERp46, we were unable to further narrow down the AdipoR1-ERp46 interaction by crosslinking.

### BACTH assay to quantify the ERp46-AdipoR1 interaction

In the previously reported heterologous expression system, the interaction between Chinese hamster ERp46 and Flag-tagged human AdipoR1 has been suggested to be mediated by a short N-terminal region of ERp46 (aa's 33-70) binding to a likewise short region of AdipoR1 (aa's 1-70) [11]. Based on this, we chose the bacterial adenylate cyclase-based two hybrid system (BACTH) to quantitatively analyze the reported AdipoR1-ERp46 interaction, as it does not depend on interaction-induced refolding of protein fragments, but rather on the co-localization of two already folded protein domains. The BACTH assay [23] uses the *Bordetella pertusis* adenylate cyclase which is not active when expressed as two fragments, T18 and T25, in the cytoplasm of *Escherichia coli* strain BTH101 lacking the *cyaA* gene encoding adenylate cyclase [13]. Enzymatic activity is restored if these fragments are brought in close proximity by fusion to interacting protein domains, such as the reported N-terminal regions of AdipoR1 (aa's 1-70) and ERp46 (aa's 33-70) fused to the C- or N-termini of T18 and T25. The activated bacterial cyclic AMP signal cascade subsequently activates the lacZ promoter, and the β-galactosidase activity can be quantified. The gene sequences of the short N-terminal domains of ERp46 (aa's 33-70) and AdipoR1 (aa's 1-70) were subcloned in the plasmids pKT25, pKT25N and pUT18C. These plasmids were co-transferred in BTH101 *Escherichia coli* cells and the protein interactions assessed. Several combinations and the parallel and anti-parallel orientations for the AdipoR1 and ERp46 N-termini, as well as the appropriate negative and positive controls were analyzed. In addition, the size of the ERp46 fragment was also varied by the inclusion of a 34 aa-long GGSGLVGGSGGGSGGGSGGGSGGGSGGGSGGGST linker sequence, to equal the length of the 70 aa-long AdipoR1 fragment size. None of the vector combinations for the suggested AdipoR1-ERp46 interaction allowed detection of quantifiable interactions (Figure 3d). A minimum of three independent experiments in quadruplicate were analyzed and the positive control yielded a strong interaction signal.

## Discussion

Previous studies from our laboratory on ccRCC patients and ccRCC models *in vitro* and *in vivo* have shown that adiponectin has tumor-suppressive effects [7] and that this hormonal axis is inhibited in ccRCC secondary to hypoadiponectinemia [5] and underexpression of AdipoR1 in the tumor tissue [7]. In this study, we demonstrate ERp46's expression status in normal kidney, primary and metastatic ccRCC and show a significantly higher ERp46/AdipoR1 expression ratio in metastatic ccRCC compared to non-metastatic ccRCC. Moreover, overexpression of ERp46 promoted RCC growth *in vivo*, encouraging ERp46's suitability as a therapeutic target in RCC. We investigated the *in vivo* significance of ERp46 on RCC tumorigenesis using mouse RAG RCC cells to be able to determine a direct effect of changed ERp46 levels without significant variability in the adiponectin signal transduction axis. All mice had equivalent levels of serum adiponectin and tumor AdipoR1 (data not shown). While the use of human RCC cells would have been preferable, we would have had to supply human adiponectin exogenously, as human receptors only partially crossreact with murine adiponectin. Our study is the first to show differential expression of ERp46 in human ccRCC and to demonstrate an effect of ERp46 expression on *in vivo* tumorigenesis potentially through increased angiogenesis (Figure 2df), a hallmark of ccRCC pathogenesis [24]. Increased protein expression of ERp46 has been demonstrated previously in human non-small cell lung carcinoma [25] and colorectal adenoma and cancer [26] and an upregulation of ERp46 at the transcriptional level has also been shown in human cervical, gastric, lung and uterine cancer, albeit in a very limited number of samples [27]. In gastric cancer cells *in vitro*, ERp46 overexpression leads to increased proliferation and decreased apoptosis [28]. Interestingly, ERp46 has been shown to be upregulated by hypoxia in endothelial cells [27], although in non-small cell lung carcinoma cells, hypoxia does not change ERp46 expression [25]. Hypoxia is a frequent event in RCC due to the inactivation of the von Hippel-Lindau tumor suppressor gene which occurs in 60% of patients [24]. The exact functions of ERp46 are not known; it has three thioredoxin-like domains and is a member of the PDI family of oxidoreductases of which another 19 members are described in mammalian ER [10]. PDIs are ubiquitous proteins, whose activity facilitates the reduction of disulfides in other proteins by cysteine-disulfide-thiol exchange [10], [29]. The formation of disulfide bonds is reversible and might be a key element in the regulation of protein stability [10]. The substrates of ERp46 and another member of the PDI family, ERp57, partly overlap, but are largely distinct [30]. ERp57 has been shown to be involved in the antigen processing machinery [31] and its role in cancer appears contrary to ERp46's. In cervical and gastric cancer, downregulation of ERp57 is associated with more aggressive tumor behavior. Chung *et al.* have shown that loss of expression of ERp57 is strongly associated with poor prognosis in cervical cancer [32]. Similarly in gastric cancer, ERp57 is downregulated and lower tumor ERp57 expression correlates with increased depth of tumor invasion and advanced overall clinical stage of disease [33].

Specific or nonspecific off-target effects of shRNA-mediated knockdown of ERp46 cannot be excluded, however, we used shRNA to knockdown ERp46 which is known to result in less off-target effects [34] compared to siRNA and we did not observe toxic or adverse effects *in vitro* or *in vivo*. Also, as in the murine RCC RAG cells in this study, we have observed similar effects of loss- and gain-of-function of ERp46 in human prostate cancer cells using different shRNA constructs specific for human ERp46 (unpublished data). Moreover, the fact that overexpression of ERp46 had the opposite result of ERp46 knockdown in our *in vivo* experiments lends credence to our claims. Knockdown of ERp46 led to an overall significant decrease in tumor volume of 59% and 40% resp. (Figure 2ce) comparable to the effect of the mammalian target of rapamycin (mTOR) inhibitor temsirolimus, an effective targeted treatment used clinically in metastatic RCC patients [35], in 786-O-bearing mice [36]. While targeting ERp46 alone may not be effective enough to slow tumor growth long-term clinically, combining inhibition of ERp46 with, for example, temsirolimus or receptor tyrosine kinase inhibitors might be a potential treatment strategy to maximally inhibit multiple growth pathways and to yield synergistic effects [37].

A close examination of the role of ERp46 in the AdipoR1 signaling pathway under native conditions deemed the postulated AdipoR1-ERp46 interaction questionable in RCC. While immunocytochemical staining of 786-O cells did indicate co-localization of ERp46 and AdipoR1, the co-immunoprecipitation approach using native human ccRCC 786-O cells, on the other hand, was inconclusive and the BACTH assay using the N-termini of both ERp46 and AdipoR1 fragments in the parallel or anti-parallel orientation showed no interaction signal. During co-immunoprecipitation, the composition of the buffer, its pH and salt concentrations are crucial. Recently, it has been reported that ERp46 requires its substrate peroxiredoxin to be hyperoxidized in order to form an interaction [38] and S-nitrosylation of the thioredoxin domains of ERp46 [39] may also be involved. It is conceivable that in human cells, additional proteins or conditions, such as oxidative and nitrosative stress, cellular anti-oxidative status or ER stress status, may mediate and enhance or modulate the putative interaction between ERp46 and AdipoR1. The BACTH system does not accurately reflect the situation in human cells. It involves only the postulated interaction between the N-termini [11] and does not depend on interaction-induced refolding of protein fragments, but rather on the co-localization of two already folded protein domains [14]. Possibly other domains in AdipoR1 and ERp46 are crucial to mediate the reported interaction between ERp46 and AdipoR1. Recently, peptide binding by the catalytic thioredoxin domains of ERp46 has been demonstrated [40], highlighting the possibility that ERp46's N-terminus might not be the actual site for interaction with AdipoR1. Proteomic and interactome studies for several PDI family members, including ERp46, under various physiological conditions have been reported [30], [39], [41], [42] providing additional data regarding other ERp46 interaction partners (see Table 2). ERp46 has a large number of interaction partners, but none resembles AdipoR1 or any other protein currently known to be involved in the signaling cascade of the adiponectin axis. Interestingly, most ERp46 interaction partners were identified out of the pool of proteins involved in oxidoreductive interactions (e.g. peroxiredoxin, ERo1α) and shown to form hetero-disulfide linkage with ERp46 in the cytoplasm [30]. Even though ERp46 appears to be involved in the adiponectin signaling pathway [11], pleiotropic mechanisms carried out by other interaction partners may convey this interaction. Interestingly in prokaryotes, thioredoxins help to stabilize heterologously expressed recombinant proteins [43], [44], a situation resembling the reported expression of the human FLAG-tagged AdipoR1 in the Chinese hamster ovary cells [11]. Also, after EGF stimulation in HeLa cells, changes in ERp46 protein interactions were observed [42] making it conceivable that changes due to reduced AdipoR1 expression in ccRCC [7] may also affect the cellular ERp46 protein interaction profile. Furthermore, if the interaction between AdipoR1 and ERp46 was of permanent nature, ERp46 would have been detectable, even in traces, in the same fractions as AdipoR1 in the cell surface protein extraction. Our results therefore rule out a stable interaction between AdipoR1 and ERp46 in our model system. Additionally, ERp46's broader suggested role in events at the cell surface [11] requires careful reconsideration.

**Table 2:** Comparisons of the different interaction partners of ERp46 reported.

Methods utilized	Reported interaction partners	Reference
Heterologous expression in Chinese hamster ovary cell line, co-immunoprecipitation	Q96A54 (AdipoR1), not Q86V24 (AdipoR2)	[11]
High-throughput complex fractionation and detection by tandem mass spectrometry	Q99426, Q12792, P38606, P55060, Q9H3U1, Q9NXH9, Q99426, Q12792, P38606, P55072, Q16643, Q9Y5V0, Q9UMX5, Q9UHR6, Q99426, P07900, O43172, Q9NX14, Q04323, Q9H3U1, Q6ZMI0, O43670, P38606	[41]
ERp46 mutagenesis and substrate trapping under either reducing or non-reducing conditions	Q96HE7, O00469, Q13751, P07942, P11047, P26006, P05107, P12109, P01130, P55268, P02462, P08572, Q9BZQ6, Q6P179, P22064, Q7Z443, Q12797, P14543, Q13162	[30]
Metabolic labeling with amino acid isotopologs in a high-throughput manner followed by size-exclusion chromatography, analysis by tandem mass spectrometry	GNAI3, P09972, B4DLZ8, P07195, P52209, P21980, Q13404, P61981, P27348	[42]

Given the expression of ERp46 in human RCC samples and its effect in our *in vivo* mouse model on RCC growth, ERp46 is a potential novel therapeutic target in RCC. However, we cautiously hypothesize that the tumorigenic properties of ERp46 in RCC cells may not be related to an inhibitory modulation of adiponectin's tumor-suppressive signaling, as an interaction with AdipoR1 could not be established. Future investigations will include the mechanism of action of ERp46 as a tumorigenic protein in RCC.

## Supporting Information

Table S1 **Bacterial strains and plasmids used.**
(PDF)

Table S2 **Amino acid sequence of peptides used in the BACTH assay.**
(PDF)
