# Supplementary material for: Endoplasmic Reticulum Protein ERp46 in Renal Cell Carcinoma
Source: PLoS One. 2014 Mar 3;9(3):e90389. doi: 10.1371/journal.pone.0090389 (PMC3940878; doi:10.1371/journal.pone.0090389)
Supplement: Table S1 — Bacterial strains and plasmids used. (PDF) [file pone.0090389.s001.pdf]

Table S1. Bacterial strains and plasmids used

**Strains or plasmids**	**Genotype or description**	**Reference**
Strains		
DH5α	F- 80dlacZ M15 (lacZYA-argF) U169, recA1 endA1hsdR17(rk-, mk+) phoA supE44 -thi-1 gyrA96 relA1	[12]
BTH101	F cya-854 recA1 endA1 gyrA96 thi1 hsdR17 spoT1 rfbD1glnV44 (AS); Nal^r	[13, 14]
Plasmids		
pUT18C	Amp^R, used for fusion at C-terminal end of the T18 polypeptide	[13, 14]
pKT25	Km^R, used for fusion at the C-terminal end of the T25 polypeptide	
pKTN25	Km^R, used for fusion at the N-terminal end of the T25 polypeptide	
pUT18C-ERp46$_N$	Amp^R, ERp46 aa 33-70 (NP_110437.2) fused in frame to the C-terminal end of the T18 polypeptide	This work
pUT18C-Linker-ERp46$_N$	Amp^R, (GS)-Linker fused in frame to the N-terminus of ERp46-T18 polypeptide	
pKT25-AdipoR1$_N$	Km^R, AdipoR1 aa 1-70 fused in frame to the C-terminal end of the T25 polypeptide	
pKTN25-AdipoR1$_N$	Km^R, AdipoR1 aa 1-70 fused in frame to the C-terminal end of the T25 polypeptide	
pUT18C-ZIP	Amp^R, with leucine zipper of GCN4	[13, 14]
pKT25-ZIP	Km^R, with leucine zipper of GCN4	
