# Supplementary material for: Endoplasmic Reticulum Protein ERp46 in Renal Cell Carcinoma
Source: PLoS One. 2014 Mar 3;9(3):e90389. doi: 10.1371/journal.pone.0090389 (PMC3940878; doi:10.1371/journal.pone.0090389)
Supplement: Table S2 — Amino acid sequence of peptides used in the BACTH assay. (PDF) [file pone.0090389.s002.pdf]

Table S2. Amino acid sequence of peptides used in the BACTH assay

Leucine Zipper of GCN4	LEDKVEELLSKNYHLENEVARKLKKLVGER
ERp46 N-terminus (33-70)	GGRWGARAQEAAAAAADGPPAADGEDGQDPHSKHLYTAD
Linker sequence	GGSGLVGGSGGGSGGGSGGGSGGGSGGGSGGGST
AdipoR1 N-terminus (1-70)	MSSHKGVVAQGNGAPASNREADTVELAELGPLLEEKGKRVIANPPKAEEEQTCPQEEEEEVRVLTLP
